# Branched-chain amino acids and sepsis: from pathological mechanisms to clinical interventions

**DOI:** 10.1186/s12986-025-01053-5

**Published:** 2025-12-27

**Authors:** Sa Yang, Hongjie Li, Haipeng Sun, Hongmei Gao

**Affiliations:** 1 Department of Intensive Care Unit, Key Laboratory for Critical Care Medicine of the Ministry of Health, Emergency Medicine Research Institute, Tianjin First Central Hospital, Tianjin, China; 2 NHC Key Laboratory of Hormones and Development, Tianjin Key Laboratory of Metabolic Diseases, Chu Hsien-I Memorial Hospital & Tianjin Institute of Endocrinology, Tianjin Medical University, Tianjin, 300134 China

**Keywords:** Branched-chain amino acids, Sepsis, Metabolic disorders, Sepsis-induced myopathy

## Abstract

Sepsis is a life-threatening organ dysfunction syndrome triggered by infection, characterized by profound metabolic disturbances and multisystem dysfunction throughout its clinical course. Branched-chain amino acids not only participate in protein homeostasis and energy metabolism but also regulate the pathophysiological processes of sepsis through multiple signaling pathways. This review systematically synthesizes current research advancements on BCAAs in sepsis, with particular emphasis on the correlation between BCAA metabolic alterations and clinical outcomes, as well as their impacts on nutritional metabolism, muscular homeostasis, and immune function in septic patients. Furthermore, we critically analyze the limitations of current clinical interventions involving BCAAs and propose future research directions, highlighting the necessity for in-depth exploration of precision BCAAs supplementation strategies tailored to different sepsis stages and their underlying mechanisms.

## Introduction

 Sepsis is essentially an infection-induced host response disorder characterized by systemic disorders that compromise multiple organ functions [1]. According to statistics, from 2017 to 2019, the number of deaths caused by sepsis in China each year was 806,728, accounting for ~ 13.1% of the total number of deaths [2]. Globally, sepsis causes 11 million deaths each year, accounting for ~ 20% of all deaths worldwide [3]. Global ageing, increased invasive treatments, and the spread of drug-resistant bacteria are driving up the incidence of sepsis and threatening the lives of patients [4].

The core pathophysiology of sepsis includes immune dysregulation, metabolic disorders, and multiple organ failure. Early sepsis is characterized by hyperactivated immune cells precipitating a cytokine storm. As the disease progresses, immune cells are damaged, exhausted, or undergo apoptosis due to initial excessive activation, eventually leading to a persistent state of immune dysfunction and immunosuppression [5]. Decades of research into immune disorders have not yet resulted in effective therapeutic agents that have a significant impact on patient prognosis. Metabolic network disorders participate in the pathological process of sepsis through multiple mechanisms, making metabolic pathway intervention a promising therapeutic target. The characteristic manifestations of sepsis include systemic inflammatory responses such as hyperthermia, tachycardia, and tachypnea, accompanied by significant immune activation and acute-phase protein elevation, and these pathological processes markedly increase energy requirements [6]. However, anorexia and gastrointestinal dysfunction frequently lead to clinically significant nutrient deprivation in these patients, whereas concurrent mitochondrial dysfunction exacerbates ATP synthesis deficiency, culminating in a vicious cycle of metabolic derangement [7]. The body initiates a hypercatabolic state during energy deficiency, and increased protein catabolism can result in sepsis-induced myopathy (SIM), also known as ICU-acquired myopathy, a common complication associated with sepsis, which is characterized by symmetrical muscle atrophy and respiratory and limb skeletal muscle weakness, leading to prolonged mechanical ventilation and limb dysfunction, and patients are at increased risk of early death [8].

BCAAs are collectively referred to as leucine, isoleucine and valine, are key components of most proteins and major energy substrates involved in catabolic reactions during fasting and sustained exercise [9]. In addition to serving as protein constituents and metabolic fuels, BCAAs function as critical signaling molecules. Leucine, the most extensively studied BCAAs, allosterically activates the mTORC1 kinase complex—a master regulator of cellular anabolism that coordinates protein synthesis, cell growth, and other biosynthetic processes [10, 11]. In terms of immunomodulation, BCAAs can directly promote immune cell function, help restore the damaged immune system, and improve nutritional status in cancer and liver disease [12].

Given the physiological functions of BCAAs, both as energy substrates and signaling molecules regulating muscle metabolism and immune function, this review systematically elucidates the metabolic changes in BCAAs in sepsis and their effects on muscle atrophy and immune function and explores their clinical significance and potential for intervention.

### Metabolic characteristics and physiological functions of branched-chain amino acids

**Fig. 1:**
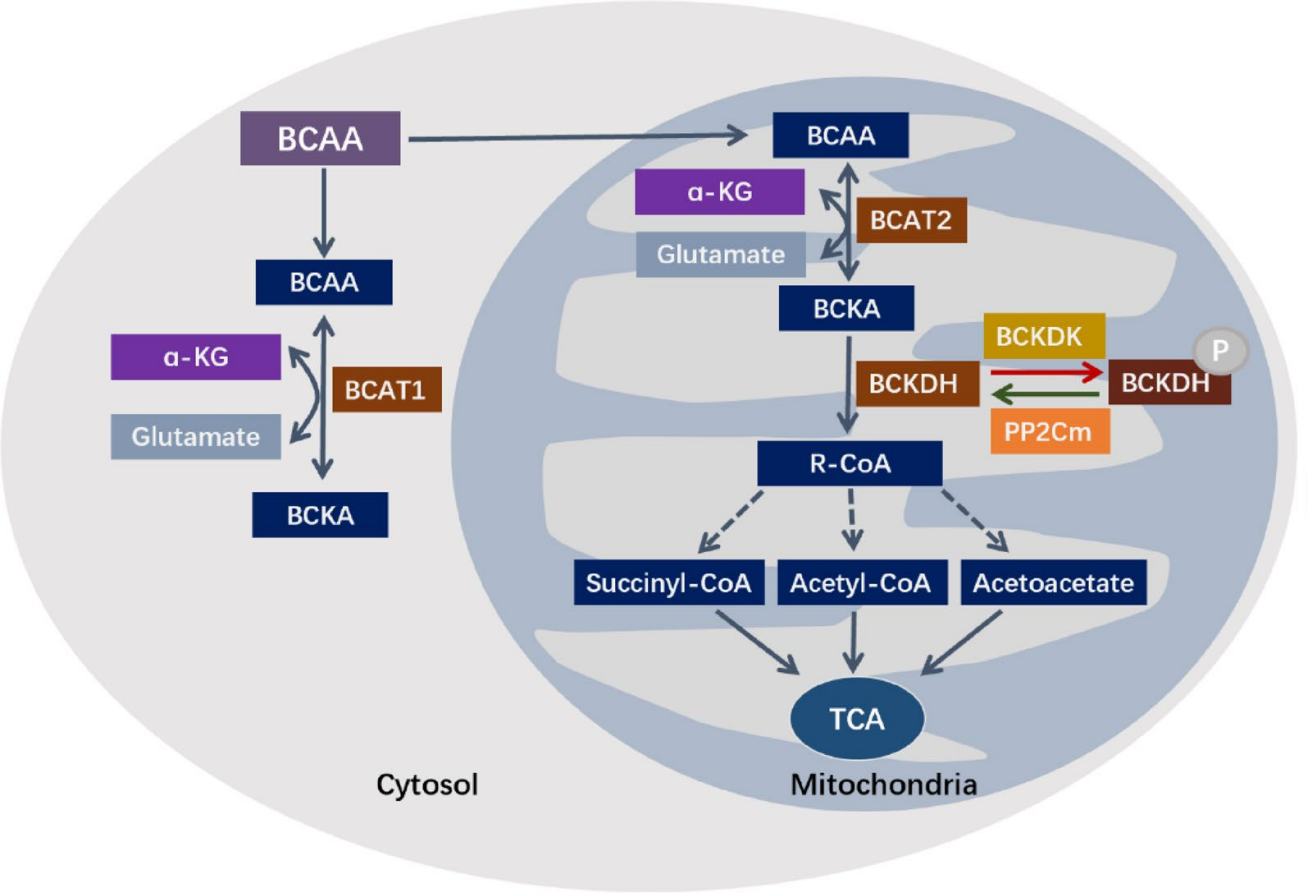
Schematic diagram of the BCAAs catabolic pathway

BCAAs are a class of essential amino acids with methyl side chains present in their structure, including leucine, isoleucine, and valine, which in mammals are derived mainly from the diet and have also been shown to be synthesized by bacteria present in the human microbiota [13]. BCAAs account for ~ 35% of the essential amino acids found in muscle proteins, and 40% of the amino acids are required for mammalian protein synthesis [14]. Whole-body metabolic tracing in mice demonstrated distinct tissue-specific BCAA oxidation patterns: skeletal muscle dominated (59% of total flux), followed by brown adipose tissue (19%), while liver, kidney and heart collectively accounted for 17% (8%, 5% and 4%, respectively) [15]. The catabolism of branched-chain amino acids (BCAAs) proceeds through three conserved enzymatic steps, with the key enzymes and regulators exhibiting distinct subcellular localizations that define the spatial organization of this pathway [16]. The initial transamination of BCAAs to their corresponding α-ketoacids (BCKAs) is catalyzed by branched-chain aminotransferases (BCATs). In humans, two BCAT isoenzymes with compartment-specific expression exist: the cytosolic BCAT1 (also known as BCATc), expressed predominantly in the brain, and the mitochondrial BCAT2 (also known as BCATm), expressed in most peripheral tissues [17, 18]. The remarkably low expression of both BCATs in the liver underlies its minimal contribution to this initial catabolic step [19]. The second, rate-limiting step involves the irreversible oxidative decarboxylation of BCKAs, which occurs within the mitochondria. This reaction is catalyzed by the branched-chain α-ketoacid dehydrogenase (BCKDH) complex, located on the inner mitochondrial membrane. The BCKDH complex comprises three core components (E1, E2, E3), among which the E1 component exists as an α₂β₂ tetramer. Specifically, E1α is encoded by the BCKDHA gene, and E1β by the BCKDHB gene [20]. The complex’s activity is dynamically regulated by a phosphorylation-dephosphorylation cycle. BCKDK, a kinase bound to the E2 subunit within the mitochondrion, inactivates the complex by phosphorylating Ser293 on the E1α subunit [21]. Conversely, the mitochondrial matrix phosphatase PP2Cm (encoded by PPM1K) activates the complex by dephosphorylating the same site [22]. Finally, the products are further metabolized via dehydrogenation reactions mediated by acyl-CoA dehydrogenases in the β-oxidation pathway [16]. The expression and activity of these catabolic enzymes display significant tissue specificity. For instance, while the expression of the inhibitory kinase BCKDK is highest in skeletal muscle, the highest BCKDH complex activity is found in the liver, with relatively lower activity in muscle, adipose tissue, and the brain [23]. For a systematic summary of the subtypes, subcellular localizations, and core functions of these BCAA metabolic enzymes, see Table 1; Fig. 1.

BCAAs are highly hydrophobic and usually form the internal core of water-soluble globular proteins together with phenylalanine and methionine, a conformation that is important for the stability of globular proteins and the maintenance of their function [24]. The catabolic products of BCAAs provide energy for the body. Leucine metabolism generates acetoacetic acid and acetyl-CoA, valine generates succinyl-CoA, and isoleucine decomposition generates acetyl-CoA and succinyl-CoA. Acetyl-CoA can enter the tricarboxylic acid cycle for oxidation and energy supply or generate ketone bodies, while succinyl-CoA can meet the body’s energy requirements through gluconeogenesis [25]. In addition, BCAAs perform diverse functions, including regulating lipid metabolism and gluconeogenesis, and serve as nitrogen donors in various biological processes. Their roles primarily involve key metabolic pathways, such as the mTORC1, AMPK, GCN2, and insulin signaling pathways [26–28].

**Table 1:** Key enzymes in BCAAs catabolism

Enzyme/ Complex	Isoforms /Subunits	Subcellular localization	Gene(s)	Primary functions
BCAT	BCAT1	Cytosol	*BCAT1*	Transaminates BCAAs to their corresponding α-ketoacids
	BCAT2	Mitochondria	*BCAT2*	Transaminates BCAAs to their corresponding α-ketoacids
BCKDH	E1α Subunit	Mitochondria	*BCKDHA*	Catalyzes the decarboxylation reaction of the BCKDH complex and mediates the regulation of its activity via the phosphorylation of Ser293
	E1β Subunit	Mitochondria	*BCKDHB*	Forms the decarboxylase component with E1α
	E2 Subunit	Mitochondria	*DBT*	As a core component of the complex, it provides a structural scaffold and catalyzes acyl-CoA synthesis
	E3 Subunit	Mitochondria	*DLD*	Oxidizes dihydrolipoic acid to produce NADH
BCKDH Kinase	BCKDK	Mitochondria	*BCKDK*	Phosphorylates and inactivates the E1α subunit of BCKDH, inhibiting BCAA catabolism
BCKDH Phosphatase	PP2Cm	Mitochondria	*PPM1K*	De-phosphorylates and activates the E1α subunit of BCKDH, promoting BCAA catabolism

### Studies on the associations of BCAAs with diseases

1. **Associations of BCAAs with diseases**

Recent studies have revealed that dysregulated BCAAs metabolism is associated with an elevated risk of multiple diseases, including insulin resistance, atherosclerosis, non-alcoholic fatty liver disease, obesity, and certain malignancies. Metabolomic data indicate that BCAAs catabolism is enhanced in obese individuals and is correlated with insulin resistance [29, 30]. BCAAs and their metabolite BCKA impair insulin signaling via mTORC1 activation. Reduced BCAAs intake effectively improves insulin sensitivity, whereas BCAAs supplementation exacerbates insulin resistance in ob/ob mice [31]. Elevated BCAAs levels resulting from impaired BCAAs catabolism upregulate lipid synthesis and suppress fatty acid oxidation through mTOR activation and AMPK signaling inhibition [32]. In cardiovascular pathophysiology, impaired BCAAs catabolism promotes heart failure by inducing oxidative stress and metabolic dysregulation during mechanical overload [33]. Chronic accumulation of BCAA caused by diet or genetic factors makes the heart prone to ischemia-reperfusion injury by exacerbating lipid peroxidation toxicity [34]. Furthermore, BCAAs catabolic metabolites serve as critical regulators of platelet activation and are correlated with arterial thrombosis risk. Targeting BCAAs catabolism or restricting dietary BCAAs intake may represent novel therapeutic approaches for the treatment of metabolic syndrome-associated thrombosis [35].

2. **BCAAs metabolism in different inflammatory states: a comparison**

BCAAs, as essential amino acids for humans, are critical for maintaining skeletal muscle protein synthesis, energy provision, and immune function. However, their metabolism is specifically disrupted in inflammatory states such as sepsis, trauma, burns, chronic inflammation with a particular focus on type 2 diabetes mellitus, and cancer cachexia, which affects inflammatory progression and disease prognosis. To clearly compare the core pathological characteristics, plasma BCAA/BCKA levels, and tissue metabolic status across different inflammatory conditions, the following table summarizes their respective features, including plasma BCAA and BCKA levels as well as tissue metabolism patterns (Table 2). 

**Table 2:** Alterations in BCAA metabolism in inflammatory diseases

	Plasma BCAA/BCKA levels	Tissue metabolic characteristics	Citations
Sepsis	Decrease in BCAA and KIC	Increased BCAA uptake but decreased oxidation in liver; increased BCAA oxidation in muscle	[36, 37]
Trauma	Decrease in BCAA	Ex vivo increase in total amino acid uptake in liver; increased BCAA oxidation in muscle	[38–41]
Burn	BCAA unchanged early stage; Decreased by 20 ~ 30% within 2 weeks in severe cases	Increased hepatic BCAA oxidation	[42–45]
T2DM	Increase in BCKA	Markedly decreased BCAA oxidation in liver, muscle, and kidney	[46]
Cancer Cachexia	Decrease in BCAA	Accelerated BCAA oxidation in muscle	[47]

3. **Individual roles of leucine**,** isoleucine**,** and valine in disease associations**

Existing studies have shown that intermittent leucine deficiency improves insulin sensitivity by sustained upregulation of GCN2 expression, a process mediated by reduced DNA methylation of hepatic GCN2 [48]. Additionally, leucine deprivation has been found to reverse chronic stress-induced depression-like behaviors by activating the signaling pathway in hypothalamic AgRP neurons; this finding identifies this hypothalamic signaling pathway as a novel therapeutic target for depression [49]. Furthermore, leucine deficiency can significantly reduce systemic fat accumulation by promoting lipid mobilization in white adipose tissue and thermogenesis in brown adipose tissue [50]. In contrast, another line of research has demonstrated that oral administration of leucine increases the risk of cardiovascular disease in obese rats, a mechanism involving enhanced glucosamine synthesis in endothelial cells and concurrent inhibition of nitric oxide production in these cells [51].

In aged mice, a low-isoleucine diet improves metabolic health, delays aging, and reduces frailty, but reduces grip strength and exerts sex-differentiated cardiac effects [52]. Dietary restriction of isoleucine alleviates high fat diet induced cognitive impairment by altering the gut microbiota, reducing neuroinflammation and insulin resistance, and improving synaptic plasticity in the mouse brain [53]. Conversely, isoleucine supplementation can also reverse mitochondrial oxidative dysfunction and protein synthesis inhibition caused by hyperammonemia, thereby improving the age-related molecular phenotype of skeletal muscle [54]. Dietary supplementation of isoleucine significantly improves colitis and growth retardation in rats by inhibiting the TLR4/MyD88/NF-κB pathway and suppressing the colonic inflammatory response [55].

Valine supplementation induces testicular apoptosis and disrupts testicular structure in mice through signaling pathways such as autophagy and RNA m⁶A methylation [56]. Long-term high dietary valine levels can improve the intestinal barrier and flora of laying hens, but inhibit fatty acid oxidation and promote adipogenesis, thereby exacerbating the development of non-alcoholic fatty liver disease [57]. As a metabolite of valine, exogenous supplementation of 3-hydroxyisobutyric acid can improve atherosclerosis by enhancing fatty acid oxidation and regulating circadian rhythms [58]. By contrast, endogenous elevation of 3-HIB is closely related to insulin resistance, fatty liver, and type 2 diabetes [59, 60].

### Pathophysiology of sepsis

Sepsis-3 defines sepsis as life-threatening organ dysfunction caused by a dysregulated host response to infection and is characterized by infection with a sequential organ failure assessment (SOFA) score ≥ 2. Septic shock is a state of circulatory failure that progresses from sepsis and is characterized by a combination of hypotension that is difficult to correct with fluid resuscitation (dependent on pressor drugs to maintain perfusion pressure) and significant lactic acidosis (>2 mmol/L) [61]. The course of sepsis exhibits complex characteristics: in the initial stage, it is characterized by the release of systemic pro-inflammatory mediators accompanied by metabolic reprogramming, which then transforms into the compensatory anti-inflammatory response syndrome (CARS) stage dominated by immunosuppression. The pathophysiological manifestations of this stage include imbalances in inflammatory regulation and activation of tissue repair [62]. Notably, immunosuppression in sepsis is a key feature of this phase, and its underlying mechanisms and biomarkers have been summarized in recent reviews [63]; however, even with early and appropriate antibiotic therapy, mortality from septic shock remains a critical challenge that requires further improvement [64]. Despite decades of research into sepsis therapeutics, extensive clinical trials of immunomodulatory agents have failed to demonstrate survival benefits—with the sole exception of activated protein C, which was subsequently withdrawn from clinical use due to safety concerns [65]. A critical limitation of current sepsis management strategies is their overemphasis on anti-inflammatory therapy, hemodynamic stabilization, and organ support while neglecting the concurrent pathogenic triad of gut microbiome dysbiosis, coagulation-complement system crosstalk dysregulation, and metabolic reprogramming.

Metabolic changes are particularly important in the pathological process of sepsis, during which immune cells use glycolysis, rather than oxidative phosphorylation, as their main source of energy under aerobic conditions [66]. Cytokine release leads to insulin resistance, and activation of the neuroendocrine system increases the release of catecholamines, promoting gluconeogenesis and glycolysis [67]. Sepsis-induced lipolysis elevation provides energy via β-oxidation of liberated fatty acids [68]. Concurrently, the “starvation-like state triggers” proteolysis upregulation, altering plasma amino acid profiles that serve as prognostic biomarkers. Targeted amino acid supplementation has therapeutic potential for improving clinical outcomes [69]. However, amino acid metabolism involves complex molecular interactions and multiple biochemical pathways, generating substantial controversy regarding the relative importance of specific amino acids, optimal therapeutic strategies, and ideal intervention timing. Notably, BCAAs—leucine, isoleucine, and valine have emerged as key players in sepsis pathophysiology.

Specifically, under physiological conditions, leucine coordinately activates GATOR2 via sensors including Sestrin2 and SAR1B. Subsequently, Rag GTPases mediate the localization and activation of mTORC1 on the lysosomal membrane, which ultimately phosphorylates S6K1 and 4E-BP1 to initiate protein synthesis [70]. During sepsis, the phosphorylation levels of mTOR and its downstream targets 4E-BP1 and S6K1 are reduced, and leucine’s capacity to activate mTOR and stimulate protein synthesis is markedly impaired [71]. Mechanistically, this injury is associated with the synergistic induction of “leucine resistance” by TNF-α and glucocorticoids in sepsis, and neutralization of TNF-α prevents sepsis-induced decreased mTOR activity and abnormal eIF4E localization [72].

**Fig. 2:**
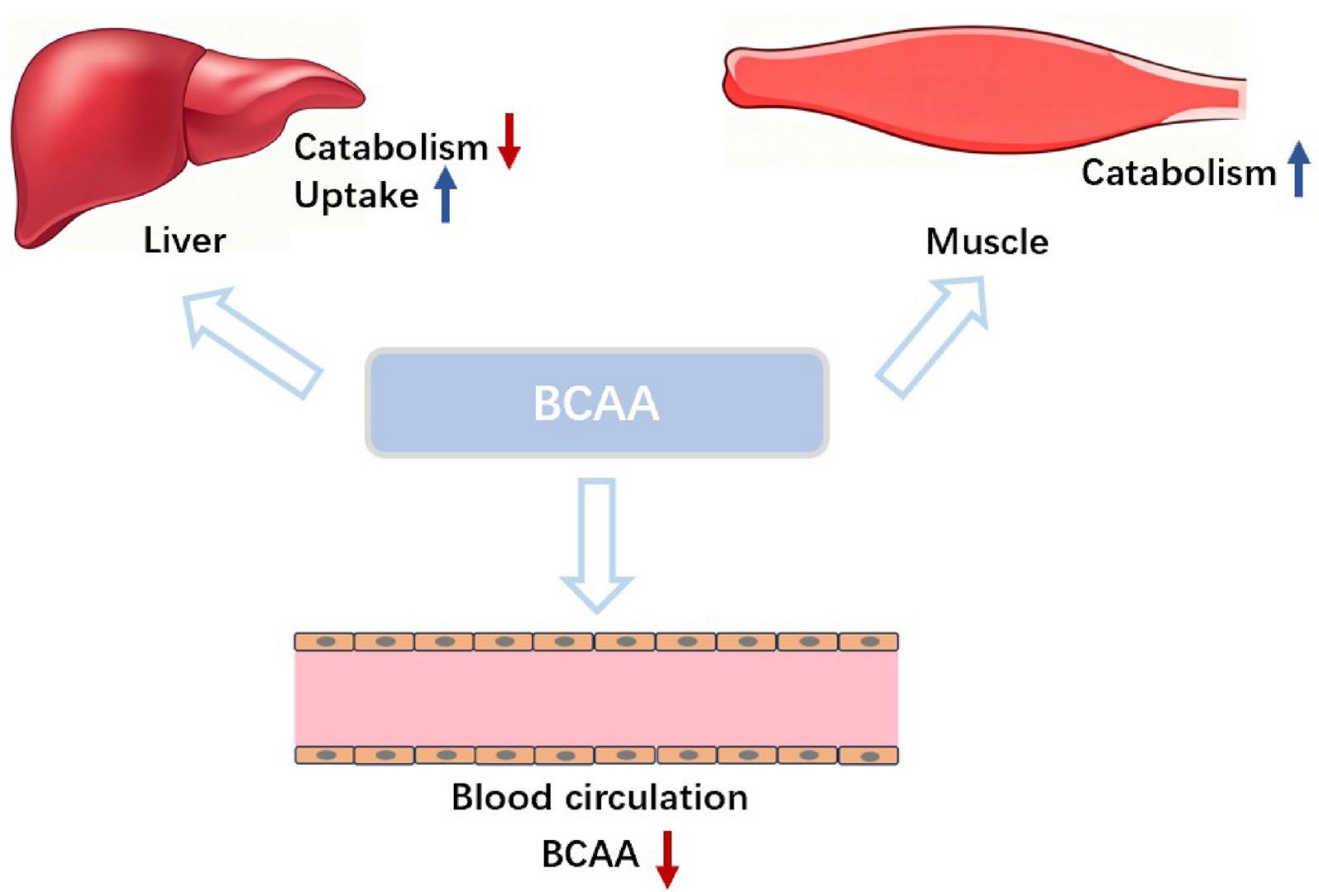
Metabolic changes of BCAAs in tissues and organs in sepsis

Meanwhile, the activity of BCKDH, a key enzyme in BCAAs catabolism, exhibits tissue-specific alterations during sepsis: hepatic BCKDH activity decreases as LPS treatment reduces α-ketoisohexanoic acid oxidation in isolated perfused rat livers [36], whereas muscle BCKDH activity increases, accompanied by enhanced BCAAs oxidation [37] (Fig. 2).

Furthermore, peroxisome proliferator-activated receptor α (PPARα) is a core regulator of BCAAs metabolism. Under physiological conditions, PPARα enhances BCKDH activity by suppressing BCKDK, and its agonist fenofibrate reduces BCAAs levels in normal rats [73, 74]. During sepsis, activation of hypoxia-inducible factor 1α (HIF1α) inhibits PPARα transcriptional activity, leading to reduced inhibition of BCKDK. This in turn inactivates BCKDH, resulting in BCAAs accumulation and impaired fatty acid oxidation. Notably, the PPARα agonist pemafibrate increases the survival rate of septic mice from 30% to 60% by activating BCKDH and promoting BCAAs oxidation [75]. Although another PPARα agonist, fenofibrate, also improves survival in sepsis models through enhanced antioxidant capacity and reduced proinflammatory cytokine levels [76, 77], whether its protective effects are linked to BCAAs metabolism remains to be elucidated. 

### Correlation between circulating BCAAs levels and sepsis prognosis

As early as the 1970 s, ion-exchange chromatography was employed for the separation and quantification of 20 standard amino acids. Freund et al. utilized an amino acid analyzer based on this technique to measure circulating BCAAs levels in septic patients, revealing significantly higher concentrations in survivors than in nonsurvivors [78]. With advancements in metabolomics technologies, liquid chromatography-tandem mass spectrometry (LC‒MS/MS) and proton nuclear magnetic resonance spectroscopy (^1^H-NMR) have surpassed traditional ion-exchange chromatography for blood metabolite detection, identification, and quantification, offering superior reproducibility and analytical stability [79]. Since then, an increasing number of studies have shown that blood BCAAs levels are strongly correlated with sepsis prognosis, e.g., Alexander et al. analyzed blood metabolite levels in ICU control patients, sepsis patients and septic shock patients via ^1^H-NMR spectroscopy and found that ICU nonsurvivors had lower blood BCAAs levels compared to survivors, and that blood BCAAs levels were a good discriminator of 28-day mortality within the ICU [80]. Research on the correlation between circulating BCAAs concentrations and sepsis survival is summarized in Table 3.

**Table 3:** Summary of studies on the correlation between BCAAs metabolic disorders and clinical outcomes in patients with sepsis

study population	Test specimens	Detection Methods	Key results	Citations
Healthy controls and sepsis	plasma	Lee employing the durum amino acid analyzer	Reduced blood BCAAs concentrations in patients with sepsis	[81]
Healthy controls and sepsis	Plasma	Biotronic LC 6001	Reduced blood BCAAs concentrations in patients with sepsis	[82]
ICU control and septic shock	Plasma	^1^H-NMR proton nuclear magnetic resonance spectroscopy	Reduced circulating BCAAs levels in patients with septic shock	[83]
ICU control and septic shock	Serum and plasma	^1^H-NMR proton nuclear magnetic resonance spectroscopy	Reduced circulating valine levels in patients with septic shock	[84]
Sepsis and no sepsis	Plasma	^1^H-NMR proton nuclear magnetic resonance spectroscopy	Valine levels in trauma patients as a predictor of sepsis development	[85]
Infected but mentally normal and altered consciousness in infectious shock	Plasma	Beckman 6300	Isoleucine levels are lower in patients with infectious shock and altered consciousness than in infected but conscious patients	[86]
Sepsis	plasma	Beckman 121-MB amino acid analyzer	Patients surviving sepsis have higher concentrations of BCAAs	[87]
Sepsis	plasma	Beckman 121-MBamino acid analyzer	Higher blood BCAAs levels in surviving sepsis patients	[78]
Normal control, sepsis and severe sepsis	Serum	high throughput mass spectrometry (HTS)	Reduced BCAAs levels in sepsis patients, and reduced BCAAs levels in nonsurvivors	[88]
ICU controls and sepsis	Serum	^1^H-NMR proton nuclear magnetic resonance spectroscopy	Lower BCAAs in nonsurvivors compared to survivors	[80]

Disruption of BCAAs metabolism is not only a pathological hallmark of sepsis but also a key driver of poor prognosis, which provides a theoretical rationale for exogenous BCAAs supplementation.

### Multiple mechanisms of BCAAs action in sepsis

1. **Nutritional regulation of BCAAs**

During sepsis, the body’s metabolic pattern undergoes significant alterations: the overall metabolic rate accelerates, accompanied by exacerbated nitrogen loss and enhanced muscle proteolysis. Concurrently, hepatic synthesis of acute-phase proteins is markedly augmented, while lipid and carbohydrate metabolism also exhibits abnormal remodeling. This hypercatabolic state, accompanied by severe disorders in amino acid metabolism, has long been regarded as an important factor affecting the mortality rate of patients with sepsis [89]. Metabolic dysregulation serves not only as a diagnostic hallmark of sepsis but also as a therapeutic target for nutritional interventions. Specifically, amino acid-targeted therapy demonstrates dual benefits in improving nitrogen balance and modulating muscle protein kinetics. Mori et al. evidenced that BCAA-enriched parenteral nutrition in septic rats significantly ameliorated nitrogen balance and peripheral cellular energy status, suggesting clinical potential for mitigating high catabolism [90]. Kawamura et al. compared the effects of 25% and 50% BCAAs parenteral nutrition solutions at a fixed ratio (isoleucine: leucine: valine = 1:1:1) on muscle protein metabolism in sepsis rats and found that 45% BCAAs significantly improved muscle mass, nitrogen balance, and the excretion of 3-methylhistidine, a marker of skeletal muscle breakdown [91]. Although preclinical research supports the metabolic benefits of BCAAs in sepsis, their clinical translational effects need to be validated through human trials. The following text systematically evaluates the existing evidence.

Metabolic disturbances in sepsis patients are not resolved by nutritional support alone, and the continued catabolism of skeletal muscle proteins is a core pathology that contributes to long-term frailty. In this context, leucine in BCAAs is a potential target for intervention because of its unique regulation of protein synthesis.

2. **Leucine modulates sepsis-induced muscle atrophy**

Sepsis-induced muscle atrophy, also known as SIM, is characterized by reduced muscle mass, loss of strength and reduced muscle fibre volume and is present in approximately 40% of critically ill patients, leading to increased ventilator use, prolonged hospitalization and mortality [92, 93]. Muscle atrophy can occur as early as the first week of sepsis and is more severe in patients with multiple organ failure compared to those with single organ failure [94]. Sepsis induces neutrophil infiltration in muscles, leading to muscle atrophy and weakness [95]. Harris et al. evaluated the strength of the adductor pollicis muscle in critically ill patients and reported severe peripheral muscle weakness, with muscle strength values as low as 30% of those in the control group [96].

The mechanisms of sepsis-induced muscle atrophy are complex, and currently identified influences include locally elevated cytokine levels, satellite cell dysfunction, free radical production, the activation of systems involving autophagy and calpain leading to increased protein hydrolysis, and a reduction in synthetic responses in muscle tissue [97]. Sepsis leads to neutrophilic infiltration of muscle tissue and elevated circulating levels of various cytokines and chemokines, such as TNF-α, IFN-γ, and IL-1β, causing atrophy and weakness [95, 98]. IL-6 is also a risk factor for septic muscle atrophy and can directly affect myogenic fibres through the gp130/JAK2/STAT3 pathway [92]. Under normal conditions, skeletal muscle is capable of self-renewal after injury, a property conferred by the presence of muscle stem cells and satellite cells; sepsis leads to long-term damage to satellite cells, affects mitochondrial function and energy metabolism, and persistently impairs muscle regeneration [99]. Excess free radicals in septic muscle tissue, including superoxide, nitric oxide, peroxynitrite, hydrogen peroxide, and hydroxyl radicals, play a key role in muscle atrophy [100]. The proteolytic mechanisms involved in muscle atrophy include the ubiquitin‒proteasome system, the autophagy‒lysosome pathway, and the calpain system [101]. The muscle atrophy box F gene (MAFbx) and muscle ring finger gene 1 (MuRF1) are muscle-specific ubiquitin ligases and key target genes for muscle atrophy [102]. In addition to the above influences, the muscle protein synthesis response is reduced in sepsis. mTORC1 affects translation initiation by regulating the formation of the eIF4F complex. mTORC1 releases 4E-BPs (eIF4E-binding proteins) by phosphorylating and inhibiting them, enabling eIF4E to bind to eIF4G and eIF4A to form the eIF4F complex, which in turn recruits the 43S preinitiation complex to the 5’ end cap structure of the mRNA and initiates translation. mTORC1 activity is inhibited in sepsis, resulting in unphosphorylated 4E-BPs, which persistently bind to eIF4E, preventing eIF4F complex formation and inhibiting translation initiation [103–105].

In the 1970 s, leucine was demonstrated to regulate muscle protein metabolism. In studies using rat diaphragms, Buse and Reid reported that increasing the extracellular leucine concentration from 0.1 mM to 0.5 mM rapidly stimulated protein synthesis while suppressing protein degradation [106]. A sepsis model was established via intraperitoneal LPS injection. LPS stimulation significantly increased skeletal muscle protein degradation, whereas dietary leucine supplementation attenuated this effect and concurrently enhanced protein synthesis [107]. Additionally, an elevation in plasma leucine levels during sepsis can enhance the assembly of active eIF4F complexes, thereby stimulating the body’s protein synthesis processes [108]. Leucine reduces mortality in mice treated with lethal doses of LPS, inhibits M1 and promotes M2 macrophage polarization, reduces proinflammatory factor levels and restores immune homeostasis [109]. Dietary leucine intake not only improves muscle tissue anabolism and restores mitochondrial respiratory capacity but also limits the proinflammatory state dominated by the systemic Th1/Th17 profile [110].In a sepsis mouse model induced by cecal ligation and puncture, leucine intervention initiated 1 h post-surgery was found to alleviate sepsis-induced skeletal muscle injury by inhibiting monocyte infiltration, reducing calpain activity, and downregulating the expression levels of inflammatory factors [111]. Leucine intervention significantly increased muscle mass and total muscle protein content in mice on day 4 after cecal ligation and puncture. Meanwhile, it downregulated the mRNA expression of muscle atrophy-related E3 ubiquitin ligases and upregulated myogenic gene expression, thus playing a key regulatory role in promoting protein synthesis and maintaining muscle mass [112]. BCATm-knockout mice studies showed enhanced skeletal muscle protein synthesis under basal conditions, with increased mTORC1 downstream 4E-BP1 phosphorylation, eIF4E・eIF4G binding, and 4E-BP1/eIF3-Raptor interaction. After LPS treatment, these mTORC1-related indices decreased significantly less than in controls. This suggests BCATm knockdown increases circulating BCAAs, improving LPS-induced catabolism of skeletal muscle protein synthesis via altered mTORC1 protein interactions [113].

In addition to leucine regulating muscle protein synthesis, its metabolite, β-hydroxy-β-methylbutyrate (HMB), plays an important role in reducing protein degradation and promoting the recovery of damaged muscle cells. LPS-induced sepsis in a rat model of sepsis was shown to reverse metabolic changes in skeletal muscle proteins by attenuating proteasomal activity and inhibiting proteolysis after HMB treatment [114]. HMB was able to block sepsis-induced activation of the 20 S proteasome subunit and double-stranded RNA-dependent protein kinase, significantly attenuating diaphragmatic muscle weakness in septic mice and preserving muscle force production at all stimulation frequencies [115].

3. **Isoleucine and immune response in sepsis**

In sepsis, isoleucine exerts a certain effect on immune function. Metabolomic profiling has shown that isoleucine interacts with hydroxybutyrylcarnitine and valerylcarnitine in septic patients, and it may serve as a potential metabolic marker reflecting disease progression [116]. Additionally, low plasma isoleucine levels are a key variable for distinguishing the severity of metabolic encephalopathy and predicting mortality outcomes; an imbalanced ratio of isoleucine to aromatic amino acids can be used to assess metabolic disorders and prognosis in sepsis [87].

In terms of immune function, isoleucine is an essential nutrient for the proliferation and functional maintenance of immune cells. From the perspective of immune cell metabolic characteristics, human immune cells possess the oxidative capacity for BCAAs, among which neutrophils specifically utilize isoleucine [117, 118], which provides a metabolic basis for isoleucine to regulate neutrophil-mediated innate immune responses. Isoleucine deficiency leads to reduced protein synthesis in immune cells due to insufficient substrates and energy, thereby inhibiting their proliferation; whereas adequate levels of isoleucine can promote the proliferation of immune cells and support their function [119]. However, there is currently a lack of reports on the effect of supplementation of isoleucine alone on immune function in sepsis, and more targeted exploration is still needed in the future to clarify this issue.

4. **Valine and intestinal injury in sepsis**

Under septic conditions, valine and its derivatives play a significant role in regulating intestinal injury. They alleviate sepsis-associated intestinal injury primarily by modulating intestinal inflammatory responses, maintaining gut microbiota homeostasis, and enhancing intestinal antibacterial capacity. The specific research evidence is as follows: In addition, γ-glutamylamino-valine reduced the expression of the proinflammatory cytokines TNF-α, IL-6 and IL-1β in plasma and small intestine in a mouse model of sepsis, which exhibited antimicrobial activity, and γ-glutamylamino-valine had an antimicrobial effect on intestinal bacterial infections [120]. Metabolomic profiling revealed reduced gut microbiota diversity and significantly decreased levels of microbiota-derived L-valine in septic patients, with L-valine levels inversely correlated with SOFA scores. Subsequent murine studies demonstrated that L-valine supplementation attenuated intestinal inflammation and injury in sepsis [121].

### Clinical evidence for BCAAs in the complementary treatment of sepsis

Although previous studies have elucidated the involvement of BCAAs in sepsis pathology through multiple pathways, including nutrient metabolism, muscle protection, and immunomodulation, the translation of these basic findings into clinical benefits still requires rigorous validation. Currently, BCAAs interventions face translational challenges from the laboratory to the clinic, including critical issues such as timing of administration, dosage optimization and patient disease severity. Furthermore, in some sepsis studies, supplementing branched-chain amino acids is beneficial for nitrogen homeostasis and improving protein metabolism, but only leucine is effective. In this section, a systematic summary of available clinical studies is presented to dissect the clinical benefit groups and potential risks of BCAAs supplementation, as shown in Table 4.

**Table 4:** Summary of clinical studies of BCAAs for complementary treatment of sepsis

Study population	Methods of intervention	Key findings	Citations
Patients with severe surgical sepsis	Two BCAAs diets: 24% and 41%	The 41% BCAAs diet induced an anabolic state in patients by day 6, providing superior therapeutic effects	[122]
Patients with sepsis due to peritonitis	Two types of BCAAs parenteral nutrition: 22.5% and 45%	45% BCAAs parenteral nutrition reduces muscle protein breakdown and replenishes muscle and visceral protein faster	[123]
Trauma and sepsis patients	Two types of BCAAs parenteral nutrition: standard and BCAA-enriched groups	Compared to the standard group, the BCAA-enriched group had significantly higher neutrophil counts but lower lymphocyte and platelet counts	[124]
Patients with sepsis	Three BCAAs for parenteral nutrition: A: 0.345 g/kg/d、B: 0.675 g/kg/d、C: 0.5 g/kg/d	Groups B and C sepsis patients have a lower mortality rate than group A	[125]
Patients with sepsis	Three types of BCAAs parenteral nutrition: 25%, 45% leucine-rich BCAAs, 45% valine-rich BCAAs	Liver protein synthesis was better in 45% leucine-rich BCAAs, but improved outcomes were not observed in the	[126]

## Conclusions

**Fig. 3:**
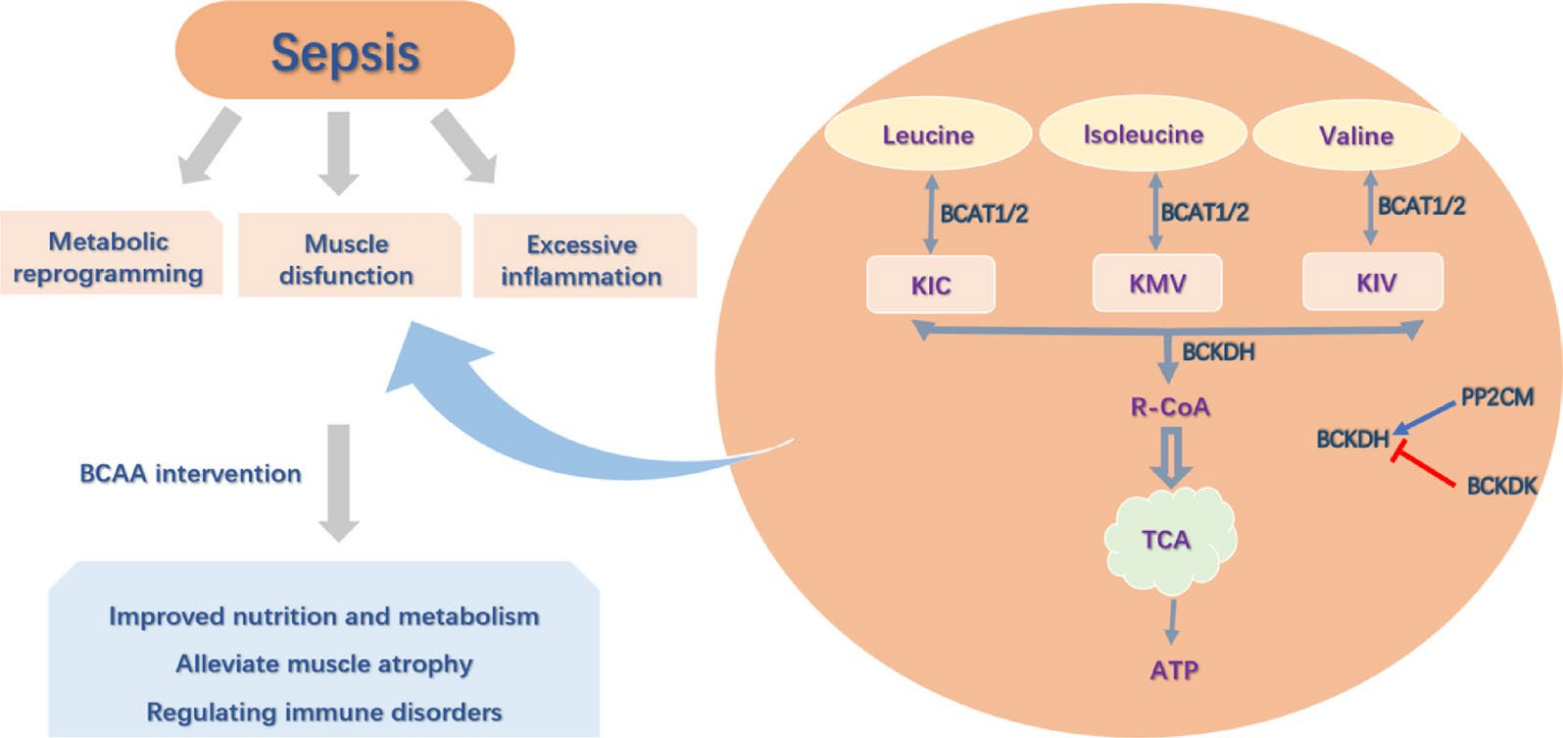
The role of BCAAs in sepsis

In summary, BCAAs exhibit therapeutic potential in sepsis, particularly in improving peripheral cellular energy metabolism, sepsis-associated muscle atrophy, and immune modulation. However, future studies should prioritize more targeted investigations, which can be advanced through the following directions: first, it is necessary to further elucidate the non-energetic functions of BCAAs, especially the molecular mechanisms by which their metabolites regulate immune function via epigenetic modifications and cellular signaling pathways Fig 3.

Second, most existing studies have focused on the effects of mixed BCAA formulations, while research on the systematic comparison of the three individual BCAAs remains to be strengthened, and this is crucial for laying the foundation for the precise treatment of sepsis. Specifically, among the three BCAAs, leucine has been relatively well studied in that it improves muscle protein synthesis and immune cell proliferation by activating the mTORC1 signaling pathway, with evidence showing it can improve muscle atrophy and metabolic disorders in sepsis. Research on isoleucine, by contrast, has mainly centered on its role as a metabolic marker and in basic nutritional support, where the ratio of isoleucine to aromatic amino acids can distinguish the severity of sepsis-associated metabolic encephalopathy and predict mortality outcomes; meanwhile, as a core nutritional substrate for macrophages, isoleucine deficiency inhibits energy production and function in immune cells, while its supplementation can restore basic immune activity, though there is currently no evidence that isoleucine can actively regulate the sepsis process through specific signaling pathways and its independent contribution to sepsis treatment remains unclear. Valine, on the other hand, exerts its core effect on intestinal protection by alleviating sepsis-associated intestinal injury through regulating intestinal inflammatory responses and maintaining gut microbiota homeostasis, thereby providing support for intestinal barrier protection in sepsis.

From a clinical perspective, significant gaps remain in understanding dose‒response relationships and the optimal timing for BCAAs interventions, particularly dynamic adjustment strategies across different sepsis stages. Future clinical research should focus on (1) patient stratification via metabolomic profiling, (2) combination therapies with other metabolic modulators, and (3) the development of targeted delivery systems for diagnostic and therapeutic applications. These advances could accelerate the translation of BCAAs research from bench to bedside, enabling precision management of sepsis.

## Data Availability

Not applicable.

## Abbreviations

ATP: Adenosine triphosphate
BCAA: Branched-chain amino acids
BCKA: Branched-chain α-keto acids
BCATs: Branched-chain amino acid aminotransferases
CARS: Compensatory anti-inflammatory response syndrome
HIF-1ɑ: Hypoxia-inducible factor-1ɑ
HMB: β-hydroxy-β-methylbutyrate
H-NMR: Proton nuclear magnetic resonance spectroscopy
ICU: Intensive care unit
IL-6: Interleukin-6
IL-1β: Interleukin-1β
LC‒MS/MS: Liquid chromatography-tandem mass spectrometry
LPS: Lipopolysaccharide
MAFbx: Muscle atrophy F-box
MODS: Multiple organ dysfunction syndrome
MuRF1: Muscle ring finger protein 1
mTOR: Mechanistic target of rapamycin
PPARɑ: Peroxisome Proliferator-Activated Receptor alpha
SIM: Sepsis-induced muscle atrophy
SOFA: Sequential organ failure assessment
TNF-α: Tumor necrosis factor-α
